# Supplementary material for: Sustained Cytotoxic Response of Peripheral Blood Mononuclear Cells from Unvaccinated Individuals Admitted to the ICU Due to Critical COVID-19 Is Essential to Avoid a Fatal Outcome
Source: Int J Environ Res Public Health. 2023 Jan 20;20(3):1947. doi: 10.3390/ijerph20031947 (PMC9915056; doi:10.3390/ijerph20031947)
Supplement: Supplementary file 1 [file ijerph-20-01947-s001.zip › ijerph-2081638-Supplemental Table S1.pdf]

Supplemental Table S1. Sociodemographic and clinical characteristics of the individuals with severe and critical COVID-19 recruited for this study at the ICU of Hospital Universitario Ramón y Cajal (Madrid, Spain) between October 2020 and April 2021.

Patient's ID	Sample (weeks)	Demographical data			Signs and symptoms								Treatment	Comorbidities			
		Age (years)	Gender	Exitus	Cough and expectoration	Dyspnea	Fever	Pneumonia	Diarrhoea and vomiting	Lethargy	Migraine	Asthenia		DM	DL	HT	DIC
**1**	0	65	F	Yes	Yes	Yes	Yes	Yes	Yes	No	No	Yes	LMWH, dexamethasone	No	Yes	No	No
	2				Yes	Yes	Yes	Yes	No	No	No	No	Dexamethasone, LMWH, antibiotics				
**2**	0	65	M	Yes	Yes	Yes	Yes	Yes	Yes	Yes	Yes	Yes	Dexamethasone, tocilizumab, antibiotics	No	No	No	No
	4				Yes	Yes	Yes	Yes	Yes	Yes	Yes	Yes	Dexamethasone, tocilizumab, antibiotics				
	6				Yes	No	Yes	Yes	Yes	Yes	No	Yes	Ceftazidime-Avibactam, Linezolid				
**3**	0	69	M	Yes	No	No	Yes	Yes	No	No	No	No	Dexamethasone, remdesivir, LMWH	No	Yes	Yes	No
	4				Yes	Yes	Yes	Yes	No	No	No	Yes	Und.		No		
	6				Yes	Yes	Yes	Yes	No	No	No	Yes	Und.		No		
**4**	0	74	M	Yes	No	No	Yes	Yes	No	No	No	No	Dexamethasone, tocilizumab	No	No	No	No
	4				No	No	Yes	Yes	No	No	No	No	Dexamethasone, tocilizumab				
**5**	0	65	M	Yes	Yes	No	Yes	Yes	Yes	Yes	No	Yes	Ceftazidime-Avibactam, Linezolid, Isavuconazol	No	No	No	No
	2				Yes	No	Yes	Yes	Yes	Yes	No	Yes	Meropenem, Linezolid, Isavuconazol				
**6**	0	56	M	Yes	No	Yes	Yes	Yes	No	No	No	No	Dexamethasone, LMWH	Yes	Yes	Yes	No
	4				Yes	Yes	No	Yes	No	No	No	No	Und.		No	No	
**7**	0	78	M	Yes	No	Yes	Yes	Yes	No	No	No	No	Ceftriaxone, Levofloxacin, Corticosteroids, LMWH	Yes	No	Yes	No
	2				No	Yes	Yes	Yes	No	No	No	No	Und.				
**8**	0	64	F	Yes	Yes	Yes	Yes	Yes	No	No	No	Yes	Piperacillin-Tazobactam, Dexamethasone, LMWH	No	No	No	No
	2				Yes	Yes	Yes	Yes	No	No	No	Yes	Meropenem, Linezolid, Dexamethasone, LMWH				
**9**	0	63	M	Yes	Yes	Yes	No	Yes	No	No	No	No	Piperacillin-Tazobactam, Dexamethasone, LMWH	No	Yes	Yes	No
	2				Yes	Yes	No	Yes	No	No	No	No	Und.				
**10**	0	56	M	Yes	No	No	Yes	Yes	Yes	No	No	No	Dexamethasone, methylprednisolone	No	Yes	Yes	No
	4				Yes	Yes	Yes	Yes	Yes	Yes	Yes	Yes	LMWH				
	8				Yes	Yes	Yes	Yes	Yes	Yes	Yes	Yes	Und.				
	10				Yes	Yes	Yes	Yes	Yes	Yes	Yes	Yes	Und.				
	11				Yes	Yes	Yes	Yes	Yes	Yes	Yes	Yes	Und.				
	12				Yes	Yes	Yes	Yes	Yes	Yes	Yes	Yes	Und.				

**11**	0	62	M	Yes	Yes	Yes	Yes	Yes	Yes	No	No	Yes	Piperacillin-Tazobactam, vancomycin	Yes	Yes	Yes	No
	2				Yes	Yes	Yes	Yes	Yes	No	No	Yes	Daptomycin, Fluconazole				
	4				Yes	Yes	Yes	Yes	Yes	No	No	Yes	Ceftazidime-Avibactam				
	5				Yes	Yes	Yes	Yes	Yes	No	No	Yes	Anidulafungin, Ceftazidime-Avibactam, Ganciclovir				
	6				Yes	Yes	Yes	Yes	Yes	No	No	Yes	Anidulafungin, Ceftazidime-Avibactam, Ganciclovir, Linezolid				
	8				Yes	Yes	Yes	Yes	Yes	No	No	Yes	Ceftazidime-Avibactam, Amikacina, Vancomycin, Levofloxacin, Voriconazol, LMWH				
	9				Yes	Yes	Yes	Yes	Yes	No	No	Yes	Und.				
	10				Yes	Yes	Yes	Yes	Yes	No	No	Yes	Und.				
**12**	0	77	M	Yes	Yes	Yes	Yes	Yes	Yes	Yes	Yes	Yes	Ceftolozano/tazobactam, tocilizumab, corticosteroids, LMWH	No	Yes	No	No
	4				No	No	Yes	Yes	No	Yes	Yes	Yes	Tocilizumab, dexamethasone, Ceftolozano/tazobactam				
**13**	0	53	M	Yes	Yes	Yes	Yes	Yes	Yes	No	Yes	No	Cefepime, Levofloxacin, Isavuconazol, LMWH	Yes	No	No	No
	2				Yes	Yes	Yes	Yes	Yes	No	Yes	No	LMWH				
**14**	0	58	M	No	Yes	Yes	Yes	Yes	No	Yes	No	Yes	LMWH, corticosteroids	No	Yes	No	No
	4				No	Yes	Yes	Yes	No	Yes	No	Yes	LMWH, daptomycin, colistin, azithromycin				
	12				No	Yes	Yes	Yes	No	Yes	No	Yes	Und.				
	13				No	Yes	Yes	Yes	No	Yes	No	Yes	Und.				
**15**	0	76	M	No	Yes	Yes	Yes	Yes	No	No	No	Yes	Piperacillin-tazobactam, LMWH , corticosteroids	No	No	No	No
	4				Yes	Yes	Yes	Yes	No	No	No	Yes	Piperacillin-tazobactam, LMWH , corticosteroids				
	8				No	Yes	Yes	Yes	No	No	No	Yes	Pentylenetetrazol				
**16**	0	56	F	No	Yes	Yes	Yes	Yes	No	Yes	Yes	Yes	Dexamethasone, LMWH, antibiotics, remdesivir	No	No	No	No
	4				Yes	Yes	Yes	Yes	No	Yes	Yes	Yes	Dexamethasone, LMWH, antibiotics, remdesivir				
	6				No	No	No	Yes	Yes	No	No	Yes	LMWH				
**17**	0	70	M	No	No	No	Yes	Yes	No	No	No	Yes	Dexamethasone, tocilizumab	No	Yes	Yes	No
	4				No	No	Yes	Yes	No	No	No	Yes	Dexamethasone, tocilizumab				
	6				Yes	Yes	Yes	Yes	Yes	No	No	Yes	LMWH				
**18**	0	62	F	No	Yes	Yes	Yes	Yes	No	Yes	Yes	Yes	Dexamethasone, antibiotics	Yes	Yes	Yes	No
	4				No	No	Yes	Yes	No	No	No	No	Und.			No	
	8				No	No	Yes	Yes	No	No	No	No	Und.			No	
**19**	0	71	F	No	Und.	Und.	Und.	Und.	Und.	Und.	Und.	Und.	Ceftriaxone, LMWH, dexamethasone	Yes	Yes	Yes	No

	2				Und.	Und.	Und.	Und.	Und.	Und.	Und.	Und.	LMWH, Piperacillin-tazobactam				
	3				Und.	Und.	Und.	Und.	Und.	Und.	Und.	Und.	LMWH				
**20**	0	50	M	No	Yes	Yes	Yes	Yes	No	No	No	Yes	Pentylenetetrazol, dexamethasone, LMWH	No	No	No	No
	2				Yes	Yes	Yes	Yes	No	No	No	Yes	Und.				
	3				Yes	Yes	Yes	Yes	No	No	No	Yes	Meropenem				
**21**	0	62	M	No	Yes	Yes	No	Yes	No	No	No	No	LMWH	No	Yes	Yes	No
	2				Yes	Yes	No	Yes	No	No	No	No	Ceftazidime Avibactam				
	6				Yes	Yes	No	Yes	No	No	No	No	Und.				
**22**	0	64	M	No	Yes	Yes	Yes	Yes	No	No	No	Yes	Dexamethasone, antibiotics, LMWH	No	No	No	No
	6				Yes	Yes	Yes	Yes	No	Yes	No	No	Und.				
**23**	0	64	F	No	Yes	Yes	Yes	Yes	Yes	Yes	Yes	Yes	Dexamethasone, LMWH, antibiotics	No	Yes	Yes	No
	4				Yes	Yes	Yes	Yes	Yes	Yes	Yes	Yes	Dexamethasone, LMWH antibiotics				

DIC: disseminated intravascular coagulation; DL: dyslipidaemia; DM: diabetes mellitus; F: female; HT: hypertension; LMWH: low molecular weight heparin; M: male; Undetermined: Und.
