# Supplementary material for: Sustained Cytotoxic Response of Peripheral Blood Mononuclear Cells from Unvaccinated Individuals Admitted to the ICU Due to Critical COVID-19 Is Essential to Avoid a Fatal Outcome
Source: Int J Environ Res Public Health. 2023 Jan 20;20(3):1947. doi: 10.3390/ijerph20031947 (PMC9915056; doi:10.3390/ijerph20031947)
Supplement: Supplementary file 1 [file ijerph-20-01947-s001.zip › ijerph-2081638-Supplemental Table S2.pdf]

Supplemental Table S2. Data of hospitalization and blood biochemistry of the individuals with severe and critical COVID-19 recruited for this study at the ICU of Hospital Universitario Ramón y Cajal (Madrid, Spain) between October 2020 and April 2021.

		**Hospitalization**						**Blood biochemistry data**										
**Patient's ID**	**Sample (weeks)**	**Hospitalization time (days)**	**Days at the ICU**	**NIV**	**IV**	**Reser voir**	**Nasal Glasses**	**CRP (mg/L)**	**LDH (U/L)**	**CK (U/L)**	**Ferritin (ng/mL)**	**IL-6 (pg/mL)**	**D-dimer (ug/mL)**	**Procalcitonin (ng/mL)**	**Lympho cytes (cells/mL)**	**Monocytes (cells/mL)**	**Platelets (cells/mL)**	**Fibrinoge n (mg/dL)**
**1**	0	42	38	No	Yes	No	No	173.6	328	-	889.97	27.8	-	0.4	1370	1260	142000	552.5
	2			No	Yes	No	No	135.3	242	-	887.97	-	-	-	1240	1390	140000	491.6
**2**	0	100	87	No	Yes	No	No	24.7	635	-	-	635.8	4045	-	870	230	277000	740
	4			No	Yes	No	No	34.8	272	-	299.46	-	-	-	1320	420	445000	740
	6			No	Yes	No	No	186	250	-	-	-	805	0.12	1910	920	776000	740
**3**	0	51	49	No	Yes	No	No	143.4	527	325	-	72.6	-	0.06	620	490	181000	-
	4			No	Yes	No	No	3.1	313	-	1076	-	-	-	1100	1130	214000	256
	6			No	Yes	No	No	191	262	-	-	-	-	-	1120	610	186000	-
**4**	0	59	59	No	Yes	No	No	18.4	238	40	521.24	-	-	-	790	810	221000	-
	4			No	Yes	No	No	100.8	411	-	-	-	-	-	1720	830	293000	740
**5**	0	100	100	No	Yes	No	No	104	295	-	-	-	-	0.04	1330	530	765000	665
	2			No	Yes	No	No	304	345	-	-	-	3307	0.14	2160	740	748000	536
**6**	0	62	16	No	No	Yes	No	55.9	345	-	2496.95	33.8	3636	2.11	900	2240	261000	484.4
	4			No	No	No	No	2.8	170	-	-	-	-	-	1290	2550	159000	-
**7**	0	22	22	No	Yes	No	No	205	-	-	-	-	2168	0.61	340	130	168000	740
	2			No	Yes	No	No	24	212	-	-	-	-	-	850	360	194000	-
**8**	0	30	29	No	Yes	No	No	202	1094	-	-	-	35000	-	860	290	468000	740
	2			No	Yes	No	No	268	739	-	-	-	1858	-	520	310	452000	740
**9**	0	16	13	No	Yes	No	No	108	13	-	-	-	1024	0.02	370	360	313000	740
	2			No	Yes	No	No	151	85	-	-	-	5172	1.38	1410	1390	195000	740
**10**	0	141	96	No	Yes	No	No	30.2	340	-	2530.05	24.8	-	-	1140	490	142000	740
	4			No	Yes	No	No	20	311	-	-	-	-	-	1320	560	269000	-
	8			No	No	No	Yes	38	548	-	-	-	-	0.06	1730	950	303000	740
	10			No	No	No	Yes	18	382	-	-	-	-	44317	1840	980	303000	683
	11			No	No	No	Yes	172	358	-	-	-	-	-	1040	620	322000	742
	12			No	No	No	Yes	17	375	-	-	-	-	0.14	2010	800	593000	497
**11**	0	101	77	No	Yes	No	No	210	562	-	-	-	-	0.97	820	330	27000	740
	2			No	Yes	No	No	2.9	595	-	-	-	7407	0.03	1490	680	221000	217
	4			No	Yes	No	No	24	415	-	-	-	1920	0.03	1120	710	205000	275
	5			Yes	No	No	No	248	496	-	-	-	-	0.35	1230	230	210000	740
	6			Yes	No	No	No	50	641	-	-	-	-	0.11	680	140	106000	184
	8			No	Yes	No	No	89	496	-	-	-	-	0.03	1020	370	290000	190
	9			No	Yes	No	No	21	-	-	-	-	-	-	1080	440	390000	381
	10			-	-	-	-	-	-	-	-	-	-	-	-	-	-	-
**12**	0	43	39	No	Yes	No	No	10.1	502	-	1254.29	386.2	9200	0.2	440	220	191000	299.3
	4			No	Yes	No	No	65.2	386	-	326.45	-	3628	0.4	1500	560	133000	558.1
**13**	0	82	74	No	Yes	No	No	267	522	15	-	-	17099	-	1090	700	96000	740

	2			No	Yes	No	No	196	279	-	-	-	-	-	820	730	235000	508
**14**	0	112	75	No	Yes	No	No	49.4	353	-	163	130	3929	0.08	1350	780	836000	601
	4			No	Yes	No	No	279	-	-	-	-	-	-	1770	870	366000	740
	12			No	Yes	No	No	67	197	-	-	-	-	-	1610	660	659000	571
	13			No	No	Yes	No	21.5	215	-	-	-	-	-	1860	750	763000	740
**15**	0	101	79	No	Yes	No	No	4.2	470	42	1333.2	1076.2	7091	0.17	2440	620	274000	375.1
	4			No	Yes	No	No	241.6	56.5	-	1024.41	-	1676	-	1910	780	632000	695.7
	8			No	No	No	Yes	-	-	-	-	-	-	-	1510	1030	335000	-
**16**	0	60	39	No	Yes	No	No	164.5	-	-	3108.58	1.09	-	0.07	740	720	426000	740
	4			No	Yes	No	No	24.3	422	-	766.07	-	-	-	840	390	242000	-
	6			No	No	No	No	4.2	256	-	-	-	-	-	1700	550	393000	370
**17**	0	73	58	No	Yes	No	No	96.8	388	27	1361.8	1258.7	-	-	630	170	72000	601.7
	4			No	Yes	No	No	124.9	380	-	1361.68	-	-	0.11	2010	580	304000	740
	6			No	No	No	Yes	8.5	237	-	-	-	-	-	3100	400	198000	197
**18**	0	79	58	No	Yes	No	No	84	372	-	-	61.4	-	-	1880	460	173000	564.6
	4			No	Yes	No	No	22.3	265	-	-	-	-	0.07	2650	630	179000	387
	8			No	No	No	No	2.2	197	-	-	-	-	-	4210	600	236000	443
**19**	0	Und	Und	No	Yes	No	No	150	423	-	-	-	17100	0.12	1040	740	294000	740
	2			No	No	No	Yes	11	248	-	-	-	2038	0.03	1440	790	307000	670
	3			No	No	No	Yes	13	-	-	-	-	-	-	1280	720	374000	-
**20**	0	23	14	No	No	Yes	No	205	615	91	5280	3.18	-	0.33	900	470	459000	740
	2			No	Yes	No	No	58	210	13	1053	52	1460	-	1730	680	332000	740
	3			No	No	No	Yes	30	285	-	-	-	-	-	1130	970	435000	-
**21**	0	29	16	No	Yes	No	No	16	365	-	-	-	-	-	1500	1810	123000	-
	2			No	No	No	No	13	393	-	-	-	-	-	1610	900	112000	231
	6			No	No	No	No	19	261	-	-	-	-	-	2370	1130	154000	307
**22**	0	74	34	No	Yes	No	No	175.3	664	-	3794.97	20.8	21973	-	990	1010	506000	740
	6			No	No	No	Yes	17.9	613	88	-	7	-	-	1830	700	457000	-
**23**	0	62	44	No	Yes	No	No	214	255	-	1469	24.4	-	-	930	300	211000	740
	4			No	Yes	No	No	127.2	407	44	629.14	-	-	0.67	2590	700	440000	-

CK: creatine kinase; CRP: C reactive protein; ICU: intensive care unit; IL-6: interleukin-6; IV: invasive ventilation; LDH: lactate dehydrogenase; NIV: non-invasive ventilation; Und: Undetermined.
